# Changes in the gene expression and methylation in chicken cecal tonsils after in ovo administration of bioactive substances

**DOI:** 10.1038/s41598-023-47080-1

**Published:** 2023-11-13

**Authors:** Aleksandra Dunislawska, Magdalena Gryzinska, Maria Siwek

**Affiliations:** 1 Department of Animal Biotechnology and Genetics, Bydgoszcz University of Science and Technology, 85-084 Bydgoszcz, Poland; 2 Institute of Biological Basis of Animal Production, Sub-Department of General and Molecular Genetics, University of Life Sciences in Lublin, 20-033 Lublin, Poland

**Keywords:** Animal biotechnology, Epigenetics

## Abstract

Cecal tonsils are the main organs which generate an immune response and also the part of the GALT, thus they are in the close proximity of the intestinal microbiota and continuously exposed to microbe-associated molecular patterns. GALT developed regulatory and anti-inflammatory mechanisms which eliminate or tolerate microbiota. Bioactive substances in ovo administration ensures an early contact between the GALT and beneficial bacteria, which greatly promotes the development of tolerance. Our previous studies have shown that the administration of bioactive substances in ovo silences gene expression in the cecal tonsils. The research hypothesis assumes that negative silencing of expression is correlated with the level of methylation in the tonsils. Therefore the current study aimed to analyze the global and gene-specific DNA methylation profiles in the cecal tonsils of two distinct chicken genotypes administered in ovo with bioactive substances. Eggs of Ross 308 and Green-legged Partridgelike were stimulated on day 12 of incubation. The injected compounds were: probiotic—*Lactococcus lactis* subsp. *cremoris*, prebiotic—galactooligosaccharides, and synbiotic—combination of both. Chickens were sacrificed on d 42 post-hatching. Cecal tonsils was collected, RNA and DNA were isolated and intended to gene expression, gene methylation and global methylation analysis. Cecal tonsils changes were observed in the methylation of 6 genes: *SYK, ANGPTL4, TNFRSF14, IKZF1, CYR61, SERPING*. Analyzes showed that the suppression of gene expression is related to the level of methylation of individual genes. Based on the results obtained in the cecal tonsils, it can be concluded that the silencing of gene expression is of an epigenetic nature. This is another study aimed at analyzing the relationship between the host, its intestinal microbiota and the possibilities of its programming.

## Introduction

Gut microbiota plays multiple roles in the growth and development of the chicken host organism. It covers the essential functions related to providing the nutrients for the host organism by participation in food digestion, functional development of the intestines. However, the gut microbiota is also a key player in maintaining a well-balanced immune system^1^. Host microbiota might be modulated with bioactive substances such as prebiotics, probiotics, synbiotics, or postbiotics. These modulations might have epigenetic effects in host organisms due to gene expression changes. Chicken development outside of the maternal organism gives the possibility to modulate gut microbiota at the very early stage of embryo development. Over the past years, our group performed many experiments on the early stimulation of chicken gut microbiota with prebiotics, probiotics or synbiotics. The summary of these experiments is presented in the review published by Siwek et al.^2^. The overall picture of these reports is as follows. Early stimulation of the gut microbiota on day 12 of egg incubation creates lifelong changes in the chicken host's intestinal morphology, immune system development, physiological characteristics, and transcriptome. The detected effects vary depending on the bioactive substances administered and the genetic background of the chickens: commercial broiler or indigenous dual-purpose chicken. Hence, one feature is common for different studies. The transcriptome of the cecal tonsils is, in most cases, down-regulated. Three various synbiotics: S1 (Raffinose family oligosaccharides (RFO) combined with *Lactococcus lactis subsp. lactis*), S2 (RFO combined with *Lactococcus lactis subsp. cremoris*) and S3 (lactose combined with *Lactobacillus acidophilus* and *Streptococcus faecium*) were administered on the 12 days of incubation of Green—legged Partidgelike eggs. Analysis of the gene expression profiles in cecal tonsils showed down-regulation of *IL-4, IL-6, IL-8, IL-12, IFN-β,* and *IFN-γ* for all experimental groups^3^. In another experiment effects of prebiotic (inulin) and synbiotic (inulin combined with *Lactococcus lactis subsp. lactis*) administered in ovo on day 12 of eggs of commercial broiler chicken were analyzed in three data points: 1, 14, and 35 days post hatch^4^. The entire panel of the immune-related genes (*IL-4, IL-6, IL-8, IL-12p40, CD80, IFN-β,* and *IFN-γ*) was downregulated in the cecal tonsils, and the magnitude increased with the age of broilers. Yet another study focused on the effects of synbiotics administered in ovo to Cobb broiler embryos proved a negative regulation of the gene expression in cecal tonsils^5^. The transcriptomic profile of *IL-12, IL-8, IL1β,* and *IFNβ* was down-regulated upon the *ovo* administration of synbiotic composed of *Lactobacillus salivarius* and GOS. Regulation of gene expression in cecal tonsils post-hatch, upon in ovo injection of inulin (prebiotic) and inulin combined with *Lactobacillus lactis* subsp. *lactis* (synbiotic) was reported by Dunislawska et al.^6^. A panel of genes: *ACOX2, BRSK2, APOA1, IRS2, APBB1IP* was analyzed on days: 1, 14, and 35 post-hatch in cecal tonsils collected from broiler chickens Ross 308. On days 14 and 35 post-hatch, the genes under the study were down-regulated in the prebiotic group (*ACOX2, BRSK2, APOA1*) and synbiotic group (*ACOX2, BRSK2, IRS2, APBB1IP*). All of the above results show a significant impact of early stimulation of gut microbiota on the expression of genes related to the immune system and basic metabolism.

Silencing of the gene expression upon early stimulation of gut microbiota with bioactive substances is most likely attributed to epigenetic mechanisms of regulation^7^. Two epigenetic mechanisms are known to be responsible for gene silencing: DNA methylation and miRNA expression. Both mechanisms were identified in genes silenced after in ovo administration of bioactive substances: prebiotics, probiotics, or synbiotics in chicken liver and spleen^8–10^. It has also been proved that the DNA methylation level is tissue, genotype, and bioactive dependent.

Therefore the current study aimed to analyze the global and gene-specific DNA methylation profiles in the cecal tonsils of two distinct chicken genotypes administered in ovo with bioactive substances.

## Results

### Gene expression in cecal tonsils

Results of gene expression analysis in cecal tonsils of two chicken genotypes are presented in Table 1. A panel of 11 genes was analyzed in three experimental groups: PRE, PRO and SYN in two genotypes: Ross broiler and GP. All of the significantly regulated genes in the SYN group were downregulated (*NFATC1, CYR61, NR4A3, SERPING1, TNFRSF14, IKZF1*) for the GP genotype. The gene expression pattern was different in Ross genotype. Two significantly regulated genes were downregulated (*CYR61, SERPING1*), and two were upregulated (*NFATC1, TNFRSF14*) after all stimulants The gene regulation effect of PRO was the least efficient for both genotypes. A single gene was upregulated in GP (*CD72*) and Ross (*NFATC1*) genotypes. The injection of PRE caused the upregulation of three genes (*NFATC1, NR4A3, TNFRSF14*), and downregulation of one gene (*CYR61*) in the cecal tonsils of Ross broilers, and the downregulation of a single gene (*CXCR5*) in GP chickens.

**Table 1:** Relative gene expression in cecal tonsils of Ross and Green-legged Partridgelike injected in ovo with probiotic, prebiotic, and synbiotic (mRNA abundance—LOG_2_ Fold Change), *P* < 0.05, a,b,c—differences between groups.

Genetic group	Ross			GP		
Substance	PRE	PRO	SYN	PRE	PRO	SYN
CD72	0.20	0.67	− 0.43	− 0.42	2.07	0.38
CXCR5	0.95	1.17	0.05	− 1.25	0.54	− 0.75
NFATC1	12.30^b^	5.93^a^	12.04^b^	− 0.91^ab^	1.07^ab^	− 0.16^ab^
SYK	0.75	0.01	1.04	0.52	0.11	0.58
CYR61	− 11.91^a^	− 4.50^b^	− 12.85^a^	− 0.19^a^	0.75^a^	− 0.50^a^
NR4A3	2.38^b^	1.37^ab^	2.40^b^	− 0.19^a^	0.76^ab^	− 0.67^a^
SERPING1	− 0.37^ab^	0.28^abc^	− 1.39^a^	− 0.72^abc^	0.44^c^	− 1.81^ab^
TNFRSF14	3.61^b^	1.58	2.45	− 0.05	1.39	− 1.32^a^
IKZF1	1.15^c^	0.65^c^	− 0.24^bc^	− 0.87^a^	− 0.27^ab^	− 2.45^a^
KLHL6	0.41^d^	0.31^c^	− 0.96^b^	− 0.35^a^	0.11	− 0.65
ANGPTL4	− 0.05	0.14	0.03	− 0.82	− 0.46	− 0.58

*PRE* prebiotic, *PRO* probiotic, *SYN* synbiotic.

### Global methylation in cecal tonsils

The results of global methylation for immune tissues are presented in Table 2. There are statistically significant differences between the probiotic, prebiotic, and synbiotic injected groups in Ross genotype. There are no significant differences between control and probiotic, nor between prebiotic and synbiotic in GP.

**Table 2:** DNA global methylation in cecal tonsils in two distinct groups of chickens—Green-legged Partridgelike (GP) and Ross. a,b—differences between groups.

Genetic group	Substance	Mean	SD	CV
ROSS	C	11.74^a^	5.66	48.22
	PRO	16.49^a^	5.75	34.86
	PRE	22.67^b^	12.12	53.45
	SYN	25.10^b^	13.8	54.96
GP	C	10.72	2.6	24.24
	PRO	14.36	6.39	44.52
	PRE	17.13	4.03	23.51
	SYN	14.36	6.39	44.52

C- control, PRO—probiotic, PRE—prebiotic, SYN—synbiotic; SD—standard deviation; CV—coefficient of variation.

### Gene-specific methylation in cecal tonsils

**Figure 1:**
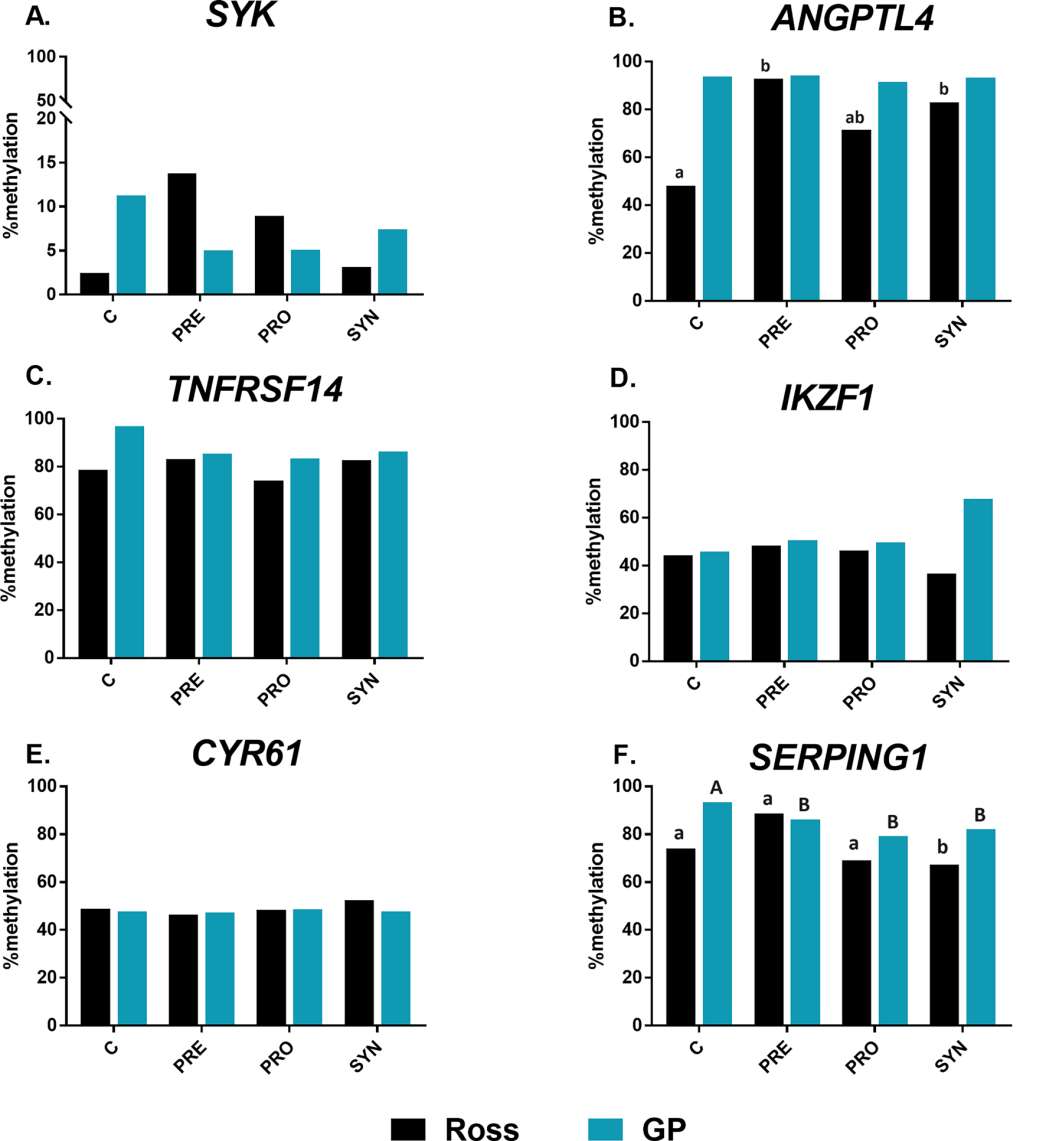
DNA methylation of the *SYK, ANGPTL4, TNFRSF14, IKZF1, CYR61, SERPING* genes in cecal tonsils. X-axis—genetic groups: Ross and Green-legged partridgelike (GP); groups: C—control, PRO—probiotic, PRE—prebiotic, SYN—synbiotic. Y-axis—the percentage of methylation. *P* < 0.05 (n = 6); (**A**, **B**)—comparison between groups in GP; a, b- comparison between groups in Ross (based on one-way ANOVA and Tukey test).

Cecal tonsils changes were observed in the methylation of 6 genes (from the panel of 11 genes): *SYK, ANGPTL4, TNFRSF14, IKZF1, CYR61, SERPING.* There is an increase in the methylation of the *SYK* gene (C group 2%) after administration of PRO (9%), PRE (14%), and SYN (3%) in Ross chicken. On the contrary, there is a decrease in the level of methylation from 11 to 7% in SYN and 5% in PRO and PRE groups in GP. *ANGPTL4* in Ross was 48% in C, increasing to 71% in PRO, 93% in PRE, and 83% in SYN in Ross. In GP methylation levels were between 91.5 and 94%. *TNFRSF14* showed a decrease in methylation for all stimulants in both genotypes, especially for the SYN in GP (decreased by 10% relative to control). The level of *IKZF1* methylation in Ross remained at the same level in all groups, while for GP it increased from 45 to 68% (SYN). The methylation level of *CYR61* gene in both GP and Ross was similar (47–48%), except for the SYN group in Ross (52%). The level of methylation of the *SERPING1* gene decreased from 93 to 79% for PRO and 82% for SYN in GP. The results are presented in Fig. 1.

An interaction analysis was performed (presented in Table 3), which showed the influence of genotype on the expression of genes: *CD72, CXCR5, NFATC1, CYR61, NR4A3, SERPING1, IKZF1 and KLHL6* and methylation of genes *SERPING1, IKZF1* and *ANGPTL4.* The substance has been shown to have an effect on expression of *NFATC1, CYR61, SERPING1* and *TNFERSF14* genes and also methylation of *SERPING1* and *ANGPTL4*. The influence of both factors on expression was noted for *NFATC1, CYR61, NR4A3, TNFSF14*, and on methylation of *SYK* and *ANTPTL4* genes.

**Table 3:** Effects of genotype and substance delivered in ovo, and their interaction on gene expression and methylation signatures in cecal tonsils of Ross and GP chickens.

Gene	Gene expression			Gene methylation		
	Genotype	Substance	Genotype x substance	Genotype	Substance	Genotype x substance
CD72	0.0001	ns	ns	ns	ns	ns
CXCR5	0.0001	ns	ns	ns	ns	ns
NFATC1	0.01	0.0001	0.0001	ns	ns	ns
SYK	ns	ns	ns	ns	ns	0.01
CYR61	0.0001	0.0001	0.0001	ns	ns	ns
NR4A3	0.0001	ns	0.01	ns	ns	ns
SERPING1	0.001	0.001	ns	0.01	0.05	ns
TNFRSF14	ns	0.01	0.01	ns	ns	ns
IKZF1	0.0001	ns	ns	0.05	ns	ns
KLHL6	0.0001	ns	ns	ns	ns	ns
ANGPTL4	ns	ns	ns	0.0001	0.001	0.001

Significance levels: *P* < 0.05; *P* < 0.01; *P* < 0.001; *P* < 0.0001 and *P* > 0.05 (non-significant, ns).

Gene expression analysis was done with RT-qPCR; gene methylation analysis was done with qMSP. The significance of effects: genotype, substance and interaction genotype x substance were calculated with two-way ANOVA (SAS Enterprise Guide 8.2 update 4; SAS Institute Inc., Cary, NC, USA).

## Discussion

Cecal tonsils are, together with the spleen, the main organs involved in immune responses. Cecal tonsils are also a part of the GALT; thus, they are close to the intestinal microbiota and continuously exposed to microbe-associated molecular patterns (MAMPs). GALT developed regulatory and anti-inflammatory mechanisms which eliminate or tolerate microbiota^11^. These mechanisms control host responses and develop tolerance to pathogens, which leads to the recognition of commensal bacteria and activation of transient and non-inflammatory immune response^12^. Synbiotics in ovo administration ensure early contact between the GALT and beneficial bacteria, which greatly promotes the development of tolerance^13^. Mucosal epithelial cells participate in the coordination of defense mechanisms which release chemokines and cytokines as a response to environmental signals. The inflammatory response can be controlled without being activated by harmless commensal gut microbiota^12^.

An impact of in ovo stimulation of gut microbiota with various bioactive (prebiotic, probiotic, synbiotic) on chicken host spleen transcriptome was already reported. A panel of 11 genes was analyzed (*CD72, CXCR5, NFATC1, SYK, CYR61, NR4A3, SERPING1, TNFRSF14, IKZF1, KLHL6, ANGPTL4*) and for 8 of them (*NFATC1, SYK, CYR61, NR4A3, SERPING1, TNFRSF14, IKZF1, ANGPTL4*) a significant regulation of gene expression was detected^9^.

The current study focuses on regulating the same panel of 11 gene expression and the gene silencing mechanism in cecal tonsils.

### Gene silencing via in ovo stimulation

Gene expression silencing after in ovo stimulation was identified, especially in genes related to immune responses. Adult chickens that received prebiotic GOS in ovo*,* showed down-regulation of 286 genes from 378 differentially expressed genes (DEGs) in cecal tonsils^14^. GOS alone and GOS-based synbiotic, and synbiotic containing inulin and *Lactococcus lactis* subsp. *lactis* caused down-regulation of *SERPING1*. This gene is responsible for regulating the complement pathway, an active part of innate immunity connecting innate and adaptive immune responses. *SERPING1* codes for a protein important to control a number of processes related to the maintenance of blood vessels, including inflammation. In our study, the level of methylation of the *SERPING1* gene decreased slightly after stimulation with probiotic and synbiotic (GOS based) in the cecal tonsils of GP compared to the control. At a basal level, it is 93% of CpG methylation, while 79–86% after stimulation. After administration of the prebiotic (GOS), there was an increase in methylation in Ross cecal tonsils.

*CYR61* is another silenced upon in ovo modulation during embryogenesis. The protein encoded by *CYR61* is a growth factor promoting endothelial cell adhesion. It also plays a role in cell proliferation, differentiation, angiogenesis, and apoptosis. In chickens infected with *Salmonella* and subsequently treated with probiotic an increased apoptosis in chicken ceca was detected. A significant role in increased apoptosis was played by *CYR61* gene^15^. In the current study, DNA methylation in SYN group of Ross genotype is related to gene expression silencing.

A third gene significantly silenced and methylated by synbiotic administration in ovo in the GP chicken is *IKZF1*. The transcription factor encoded by *IKZF1* gene belongs to the family of zinc—finger DNA -binding proteins associated with chromatin remodeling. The protein is involved in the regulation of lymphocyte differentiation. The mutation in *IKZF1* gene was liked to Marek’s disease in chickens^16^.

A similar gene silencing and methylation pattern is detected for *TNFRSTF14*. This gene encodes a tumor necrosis factor (TNF) receptor superfamily member. The encoded protein is responsible for inflammatory and inhibitory T-cell immune response activation pathways.

### Substance-dependent methylation

Probiotic—*Lactococcus lactis* subsp. *cremoris* strongly stimulates cytokine and chemokine expression in the in vitro model. Endogenous intestinal microbiota produces diet-dependent vitamins and SCFAs^17^. Bacterial metabolites such as butyrate form the basis of food for intestinal epithelial cells—colonocytes. Bacterial metabolites also affect the host cell epigenome, thereby regulating gene expression^18^. The effect of probiotics on DNA methylation has been proven many times in mammalian studies. Vahamiko et al. showed that *Lactobacillus* and *Bifidobacterium*—based probiotic supplementation led to a decrease in the level of DNA methylation in 37 gene promoters and an increase in the level of one promoter in women^19^. Zhang et al. showed that *Bifidobacterium* supplementation modifies the level of methylation in the *FOXP3* gene promoter (a marker of regulatory T cells) in colitis rats, significantly demethylating several CpG sites in the gene promoter^20^.

Administration of synbiotic in ovo, causes significant changes in methylation levels in cecal tonsils GP in the *TNRFSF14* and *IKZF1* genes. Injection in ovo of prebiotic or probiotic alone does not have such an impact on the host organism. The administered synbiotic exhibits a synergistic interaction between the prebiotic and probiotic components in vitro and possesses immunomodulatory potential against host cells^21^.

### Genotype-dependent methylation

Global methylation analysis showed apparent differences between two distinct chicken genotypes equally stimulated in ovo*.* The pattern of the genotype-dependent methylation in cecal tonsils is the same as already detected in spleen^9^. Despite the same substances used in ovo stimulation and identical rearing conditions of both genotypes (GP and Ross), methylation was detected only in Ross broilers. The differences between the two genotypes are present at all levels. GP is a dual-purpose chicken, kept in a closed population for many generations. This breed is considered to be a dual–purpose type of chicken, resistant to harsh environment^22^. On the other hand, Ross broiler is a meat-type chicken under intense selection pressure.

Obtained results are in line with the research on humans and model organisms, which show that DNA methylation depends on the genotype, the environment, and the interaction between them^23^.

Further steps in the context of silencing methylation-dependent gene expression in poultry should include an analysis of the impact of various substances administered in ovo, in particular other substances, to be able to analyze the impact of given groups of substances. Undoubtedly, further research should be directed towards higher throughput methods of methylation analysis.

## Materials and methods

### Experimental setup and tissue collection

The experimental setup and tissue collection were previously described in Dunislawska et al.^9^ and Dunislawska et al.^24^. 600 eggs of Ross 308 broiler chicken and 600 eggs of Green-legged partridgelike were incubated in standard conditions. On day 12 of incubation, eggs were randomly distributed into experimental groups (150 eggs per group): (1) probiotic (PRO)—*Lactococcus lactis* subsp. *cremoris*, (2) prebiotic (PRE)—galactooligosaccharides (GOS; Bi2tos; Clasado Biosciences, Ltd., Reading, UK) (3) synbiotic (SYN)—*Lactococcus lactis* subsp. *cremoris* with GOS. The set amount of bacteria was 10^5^ bacteria CFU egg^−1^. The amount of prebiotic was 3.5 mg egg^−1^. The control group (C) was injected with 0.2 mM physiological saline (0.9%). Eggs were injected into an air chamber with 0.2 mL of aqueous solution of each substance. After hatching, birds were housed in litter pens (4 replicates/group, 8 animals each) for 42 days. Feeding and thermal conditions are described in Dunislawska et al.^24^. Six randomly selected individuals from each group were sacrificed by decapitation (cut between the first cervical vertebra and the occipital condyle) on day 42 post-hatching. Before slaughter, the birds were stunned (concussion/blow to the head) following Dz. U. I. 303 of 18 November 2009 (Methods of killing animals).

### RNA and DNA isolation

Cecal tonsils (n = 6/group) were fixed in RNA stabilization buffer for RNA isolation (fix RNA, EURx, Gdansk, Poland). RNA extraction was prepared using TRI reagent (MRC, Cincinnati, USA) and a commercial kit for RNA purification (Universal RNA Purification Kit, EURx, Gdansk, Poland). Tissue were homogenized using the TissueRuptor homogenizer (Qiagen GmbH, Hilden, Germany) in TRI reagent. DNA from cecal tonsils was isolated using the phenol–chloroform method^25^ (procedure described in Dunislawska et al.^10^). DNA and RNA quality and quantity was checked by electrophoresis (2% agarose gel) and spectrophotometry (NanoDrop2000, Scientific Nanodrop Products, Wilmington, USA). RNA from cecal tonsils was intended for gene expression analysis (RT-qPCR), while DNA was intended for global methylation and gene-specific methylation (qMSP).

### Gene expression analysis

Gene expression analysis was performed as descripted in Dunislawska et al. (2021)^9^ by quantitative reverse transcription PCR (RT-qPCR). cDNA was synthesized using Maxima First Strand cDNA Synthesis Kit for RT-qPCR (Thermo Scientific/Fermentas, Vilnius, Lithuania), following the manufacturer's recommendations. The qPCR reaction mixture included Maxima SYBR Green qPCR Master Mix (Thermo Scientific/Fermentas, Vilnius, Lithuania), one μM of each primer, and diluted cDNA (140 ng). Thermal cycling was performed in a LightCycler II 480 (Roche Diagnostics, Basel, Switzerland) and consisted of: initial denaturation at 95 °C for 15 min, 40 cycles of amplification (denaturation at 95 °C for 15 s, annealing at 58 °C for 20 s, and elongation at 72 °C for 20 s) and melting curve. The annealing temperature was 58 °C for all target genes. Fluorescence was measured at the end of each elongation step. The melting curve was generated by increasing the temperature in increments up to 98 °C and measuring the fluorescence of the melting amplicon. Each reaction was conducted in two technical replicates. The panel of genes was selected based on microarray data. The selection and primer sequences protocol were described in Dunislawska et al.^9^. Relative gene expression analysis was conducted separately for each experimental group by the ΔΔCt method^26^ using *ACTB*^27^ and *G6PDH*^28^ as reference genes (geometric means of cycle threshold values of reference genes). Statistical analysis was carried out by SAS Enterprise Guide 8.2 (SAS Institute Inc., Cary, NC, USA. The quantitative values (Ct values) were first analyzed for normality using the Shapiro—Wilk test. The homogeneity of variance test (Levene’s test) was also carried out. The one-way ANOVA and Tukey test were used (*P* < 0.05).

### Global methylation analysis

According to the manufacturer's protocol, global DNA methylation analysis was prepared using a commercial kit for methylated DNA quantification (MDQ1, Imprint Methylated DNA Quantification Kit, Sigma-Aldrich). DNA isolated from cecal tonsils (n = 6) was diluted in a binding solution to a final concentration of 150 ng/µl. The absorbance was measured at 450 nm. Six samples, each derived from a different individual, were analyzed for each group. The absorbance measurements are based on two technical repeats. Global DNA methylation level was calculated using the following formula: $\frac{{A}_{450}S-{A}_{450}B}{{A}_{450}MC-{A}_{450}B} x 100\%$, where ${A}_{450}S$ is the average absorbance of the sample, ${A}_{450}B$ is the average absorbance of the blank, ${A}_{450}MC$ is the average absorbance of the methylated control. Statistical analysis was carried out by using SAS statistical software and was performed using the general linear model (GLM procedure) and Duncan test.

### Gene-specific methylation analysis (qMSP)

The isolated DNA was subjected to methylation analysis using the qMSP method described in Dunislawska et al.^10^. According to the manufacturer's instructions, the conversion was carried out using the EpiJet Bisulfite Conversion Kit (Thermo Fisher Scientific/Fermentas, Vilnius, Lithuania). qPCR reaction was performed for the panel of genes and also applied in gene expression analysis (primer design method and sequences based on Dunislawska et al.^9^. The qPCR analysis was performed in LightCycler 480 (Roche Diagnostics, Risch-Rothreuz, Switzerland) thermal cycler. The reaction mixture contained the Maxima SYBR Green qPCR Master Mix intercalating dye (Thermo Fisher Scientific/Fermentas, Vilnius, Lithuania). After amplification, a melting curve was generated for each product (n = 6/group). The relative level of DNA methylation [%] was calculated based on the results of melting curves (read fluorescence level) for each individual according to the formula^29^: $\% of methylation=100 x\left( \frac{M}{M+U}\right),$ where M—average fluorescence intensity of the methylated product, U—average fluorescence intensity of the unmethylated product. A one-way ANOVA and Tukey test was performed (comparison of all groups; *P* < 0.05) by using SAS statistical software. Interaction analysis for both gene expression and gene methylation was performed. The significance of effects: genotype, substance and interaction genotype × substance were calculated with two-way ANOVA by using SAS statistical software.

### Ethics approval and consent to participate

The experiment was approved by the Local Ethics Committee for Animal Experiments (Bydgoszcz, Poland) (study approval reference number 16/2014). All methods were carried out in accordance with relevant guidelines and regulations.

## Data Availability

All data are available from the corresponding author (Dunislawska Aleksandra) of the manuscript.

## Abbreviations

mRNA: Messenger RNA
GOS: Galaktooligosaccharides
P1: GOS
P2: Inulin
S1: *Lactococcus lactis* Subsp. *lactis* + inulin
S2: *Lactobacillus salivarius* + GOS
S3: *Lactococcus lactis* Subsp. *cremoris* + GOS
FC: Fold change
GP: Green-legged partridge
Ross: Ross308 broiler chicken
PRO: Probiotic
PRE: Prebiotic
SYN: Synbiotic
RT-qPCR: Reverse transcriptionquantitative polymerase chain reaction
qMSP: Real-time quantitative methylation-specific polymerase chain reaction
Ct: Cycle threshold
M: Methylated/average fluorescence intensity of the methylated product
U: Unmethylated/average fluorescence intensity of the unmethylated product
F: Forward
R: Reverse
SD: Standard deviation
CV: Coefficient of variation
LTA: Lipoteichoic acid
LPS: Lipopolysaccharide
ACTB: Beta actin
G6PDH: Glucose-6-phosphate dehydrogenase
CD72: CD72 molecule
CXCR5: C-X-C chemokine receptor type 5
NFATC1: Nuclear factor of activated T-cells 1
SYK: Tyrosine-protein kinase
CYR61: Cysteine-rich angiogenic inducer 61
NR4A3: Nuclear receptor subfamily 4 group A member 3
SERPING1: Serpin family G member 1
TNFRSF14: TNF receptor superfamily member 14
IKZF1: IKAROS family zinc finger 1
KLHL6: Kelch like family member 6; ANGPTL4: Angiopoietin-like 4
GLM: General linear model

## References

[1] Zheng D, Liwinski T, Elinav E (2020). Interaction between microbiota and immunity in health and disease. Cell Res..

[2] Siwek M (2018). Prebiotics and synbiotics—in ovo delivery for improved lifespan condition in chicken. BMC Vet. Res..

[3] Sławinska A, Siwek MZ, Bednarczyk MF (2014). Effects of synbiotics injected in ovo on regulation of immune-related gene expression in adult chickens. ajvr.

[4] Płowiec A, Sławińska A, Siwek MZ, Bednarczyk MF (2015). Effect of in ovo administration of inulin and *Lactococcus lactis* on immune-related gene expression in broiler chickens. Am. J. Vet. Res..

[5] Dunislawska A (2017). Synbiotics for broiler chickens—in vitro design and evaluation of the influence on host and selected microbiota populations following in ovo delivery. PLoS ONE.

[6] Dunislawska A (2021). Molecular response in intestinal and immune tissues to in Ovo administration of inulin and the combination of inulin and *Lactobacillus lactis* Subsp. cremoris. Front. Vet. Sci..

[7] Dunislawska A, Slawinska A, Siwek M, Bednarczyk M (2021). Epigenetic changes in poultry due to reprogramming of the gut microbiota. Anim. Front..

[8] Sikorska M, Siwek M, Slawinska A, Dunislawska A (2021). miRNA profiling in the chicken liver under the influence of early microbiota stimulation with probiotic, prebiotic, and synbiotic. Genes.

[9] Dunislawska A, Slawinska A, Gryzinska M, Siwek M (2021). Interaction between early in ovo stimulation of the gut microbiota and chicken host—splenic changes in gene expression and methylation. J. Anim. Sci. Biotechnol..

[10] Dunislawska A, Slawinska A, Siwek M (2020). Hepatic DNA methylation in response to early stimulation of microbiota with *Lactobacillus* synbiotics in broiler chickens. Genes.

[11] Brisbin JT (2008). Gene expression profiling of chicken lymphoid cells after treatment with *Lactobacillus acidophilus* cellular components. Dev. Comp. Immunol..

[12] Galdeano CM, de Moreno de LeBlanc A, Vinderola G, Bonet MEB, Perdigón G (2007). Proposed model: Mechanisms of immunomodulation induced by probiotic bacteria. Clin. Vaccine Immunol..

[13] Sansonetti PJ, Di Santo JP (2007). Debugging how bacteria manipulate the immune response. Immunity.

[14] Slawinska A, Plowiec A, Siwek M, Jaroszewski M, Bednarczyk M (2016). Long-term transcriptomic effects of prebiotics and synbiotics delivered in ovo in broiler chickens. PLoS ONE.

[15] Higgins SE, Wolfenden AD, Tellez G, Hargis BM, Porter TE (2011). Transcriptional profiling of cecal gene expression in probiotic- and Salmonella- challenged neonatal chicks. Poult. Sci..

[16] Steep A (2022). Identification and validation of Ikaros (IKZF1) as a cancer driver gene for Marek’s disease virus-induced lymphomas. Microorganisms.

[17] Greer JB, O’Keefe SJ (2011). Microbial induction of immunity, inflammation, and cancer. Front. Physiol..

[18] Alenghat T, Artis D (2014). Epigenomic regulation of host-microbiota interactions. Trends Immunol..

[19] Vähämiko S (2019). The impact of probiotic supplementation during pregnancy on DNA methylation of obesity-related genes in mothers and their children. Eur. J. Nutr..

[20] Zhang M (2017). Bifidobacterium longum affects the methylation level of forkhead box P3 promoter in 2, 4, 6-trinitrobenzenesulphonic acid induced colitis in rats. Microbial. Pathog..

[21] Sławińska A, Siwek M, Bednarczyk M (2016). In vitro screening of immunomodulatory properties of synbiotics in chicken DT40 cell line. Anim. Sci. Pap. Rep..

[22] Siwek M (2013). Insights into the genetic history of Green-legged Partridgelike fowl: mtDNA and genome-wide SNP analysis. Anim. Genet..

[23] Berbel-Filho WM, Rodríguez-Barreto D, Berry N, Garcia De Leaniz C, Consuegra S (2019). Contrasting DNA methylation responses of inbred fish lines to different rearing environments. Epigenetics.

[24] Dunislawska A, Pietrzak E, Wishna Kadawarage R, Siwek M (2023). MicroRNA expression in immune tissues of adult chickens after embryo stimulation with bioactive substances. Sci. Rep..

[25] Maniatis, T., Fritsch, E. & Sambrook, J. Molecular cloning: A laboratory manual. Cold Spring Harbor Laboratory. Cold Spring Harbor Laboratory, Cold Spring Harbor, NY, pp. 507–520 (1982).

[26] Livak KJ, Schmittgen TD (2001). Analysis of relative gene expression data using real-time quantitative PCR and the 2(-Delta Delta C(T)) Method. Methods (San Diego, Calif.).

[27] Sevane N (2014). Dietary inulin supplementation modifies significantly the liver transcriptomic profile of broiler chickens. PLoS ONE.

[28] De Boever S, Vangestel C, De Backer P, Croubels S, Sys SU (2008). Identification and validation of housekeeping genes as internal control for gene expression in an intravenous LPS inflammation model in chickens. Vet. Immunol. Immunopathol..

[29] Fackler MJ (2004). Quantitative multiplex methylation-specific PCR assay for the detection of promoter hypermethylation in multiple genes in breast cancer. Cancer Res..

